# Supplementary material for: Effects of inbound attendees of a mass gathering event on the COVID-19 epidemic using individual-based simulations
Source: PLoS One. 2025 Apr 23;20(4):e0321288. doi: 10.1371/journal.pone.0321288 (PMC12017503; doi:10.1371/journal.pone.0321288)
Supplement: Attachment — Submitted filename: PONE-D-23-04678_R1.pdf [file pone.0321288.s002.pdf]

PLOS ONE

Effects of inbound attendees of a mass gathering event on the COVID-19 epidemic using individual-based simulations

--Manuscript Draft--

**Manuscript Number:**	PONE-D-23-04678R1
**Article Type:**	Research Article
**Full Title:**	Effects of inbound attendees of a mass gathering event on the COVID-19 epidemic using individual-based simulations
**Short Title:**	Individual-based simulations of inbound attendees in a mass gathering event amid a pandemic
**Corresponding Author:**	Masaya Saitoh<br>Nagasaki Kenritsu Daigaku<br>JAPAN
**Keywords:**	Individual based simulation; mathematical model of infectious diseases; COVID-19
**Abstract:**	Given that mass gathering events involve heterogeneous and time-varying contact between residents and visitors, we sought to identify possible measures to prevent the potential acceleration of the outbreak of an emerging infectious disease induced by such events. An individual-based simulator was built based on a description of the reproduction rate among people infected with the infectious disease in a hypothetical city. Three different scenarios were assessed using our simulator, in which controls aimed at reduced contact were assumed to be carried out only in the main event venue or at subsequent additional events, or in which behavior restrictions were carried out among the visitors to the main event. The simulation results indicated that the increase in the number of patients with COVID-19 could possibly be suppressed to a level equivalent to that if the event were not being held so long as the prevalence among visitors was only slightly higher than that among domestic residents and strict requirements were applied to the activities of visitors.
**Order of Authors:**	Masaya M Saito
	Kotoe Katayama
	Akira Naruse
	Peiying Ruan
	Michio Murakami
	Tomoaki Okuda
	Tetsuo Ysutaka
	Wataru Naito
	Masaharu Tsubokura
	Seiya Imoto
**Response to Reviewers:**	Reply to Reviewer 1<br>Could you please provide further detail on how the individual based simulator was built and the specific assumptions used in the model?<br>Can you elaborate on the reasons behind the choice of the hypothetical city used in the simulation?<br>[answer] Our main interest is to assess whether additional gathering accompanying the main event enhance the reproduction. Emphasizing this point, we have rephrased the paragraphs in section 2.1 Individual-based model.<br><br>Were any limitation or assumptions made regarding the potential spread of the infectious disease beyond the event and the city?<br>[answer] In this study, we haven't discussed in detail how degree people are willing to corporate the recommendation. Along with your next remark, we have add a discussion section for this topic.

	While the results of your study suggest that the increase in the number of COVID-19 patients can be suppressed to a level equivalent to that if the event were not being held, can you discuss any potential challenges or limitations to implementing the suggested controls and restrictions on visitors?<br>[answer] The controls discussed in this work are voluntary-basis and their effectiveness depends on how degree the participants are willing to cooperate. We refer several articles which do not directly evaluate this but would help us to discuss this problem and add the sentences below:<br>The controls discussed in this work are voluntary-basis and hence the degree people are willing to cooperate limits the effectiveness. People yielding higher score in willingness to cooperate may not show a greater acceptance of behavioral regulations [17].<br>The cultural difference affects on which of individual-basis or law-enforced measures are preferable. A comparative study of three countries [18] shows Chinese participants are more acceptable to enforced measures than Japan and US, while US and Chinese more accept individual-level measures than does Japanese. Another investigation [19] supports that Japanese people are reluctant to enforced restrictions in a greater degree than the other participating countries.<br>As for mask wearing, one of self-motivated measures, there are studies over 50 US states and over the world that support people in collectivistic communities more frequently wear masks than in individual ones [20].<br>Also, it is possible to build a cooperative relationship among supports fan, academic experts, and event organizers: as high as about 98% of chant cheering participants wore masks in games held by Japan Football leagues [21].<br><br>Replay to Reviewer 3<br><br>2) A mass gathering event can be superspreading events (SSE) and it has a fairly formal definition in infectious disease epidemiology literature, first requiring an estimate of R, and then identifying events where the number of secondary infections from a single case is substantially above the average (i.e. substantially above R), a minimum threshold might be at least 2x R or 3x R. In this regard, I would like you to read on this paper and cite to further improve the introduction and discussion sections, https://doi.org/10.1080/03007995.2022.2125258<br><br>3) Additionally, the authors may also use these papers in describing and citing SSEs from other countries, such as what happened in India (the Kumbh Mela massive SSE which infected millions of people) https://doi.org/10.4269/ajtmh.21-0601 and in Thailand (the scattered SSEs during holidays) https://doi.org/10.34172/ijtmgh.2021.33<br>[answer] Thank you for giving a remark on the importance of superspreading and related references. To address your suggestion, we have added a topic addressing your suggestion on superspreading in Introduction.<br><br>4) It is also nice to include some recommendations and to further improve the conclusion.<br>[answer] I have added a paragraph in Conclusion section to discuss how degree voluntary based measures are accepted. The Kumbh Mela massive SSE case is again referred here as a case study where a cooperation among participants is not well established.<br><br><br><br>Changes in reference list<br><br>I below show the changes in the reference list. The number to each reference is of the present version (i.e. renumbered from the original submission).<br>Added articles in this revision: 7, 8, 9, 10, 11, 12, 17, 18, 19, 20, and 21.<br>Removed articles in this revision: none.<br>Articles whose reference is modified: 26<br>Reason: The original URL in a governmental web site is no longer available and replaced to wayback machine’s URL.
**Additional Information:**	

Powered by Editorial Manager® and ProduXion Manager® from Aries Systems Corporation

Question	Response
**Financial Disclosure**<br><br>Enter a financial disclosure statement that describes the sources of funding for the work included in this submission. Review the submission guidelines for detailed requirements. View published research articles from *PLOS ONE* for specific examples.<br><br>This statement is required for submission and **will appear in the published article** if the submission is accepted. Please make sure it is accurate.<br><br>**Unfunded studies**<br>Enter: *The author(s) received no specific funding for this work.*<br><br>**Funded studies**<br>Enter a statement with the following details:<br>• Initials of the authors who received each award<br>• Grant numbers awarded to each author<br>• The full name of each funder<br>• URL of each funder website<br>• Did the sponsors or funders play any role in the study design, data collection and analysis, decision to publish, or preparation of the manuscript?<br>• **NO** - Include this sentence at the end of your statement: *The funders had no role in study design, data collection and analysis, decision to publish, or preparation of the manuscript.*<br>• **YES** - Specify the role(s) played.<br><br>* typeset	One of the authors, Masaya M. Saito was supported by JSPS KAKENHI Grant Numbers JP18K11541. This funder had no role in study design, data collection and analysis, decision to publish, or preparation of the manuscript.
**Competing Interests**<br><br>Use the instructions below to enter a competing interest statement for this submission. On behalf of all authors, disclose any competing interests that could be perceived to bias this work—acknowledging all financial support and any other relevant financial or non-financial competing interests.	The authors have declared that no competing interests exist.

This statement is required for submission and **will appear in the published article** if the submission is accepted. Please make sure it is accurate and that any funding sources listed in your Funding Information later in the submission form are also declared in your Financial Disclosure statement.<br><br>View published research articles from *PLOS ONE* for specific examples.<br><br>**NO authors have competing interests**<br><br>Enter: *The authors have declared that no competing interests exist.*<br><br>**Authors with competing interests**<br><br>Enter competing interest details beginning with this statement:<br><br>*I have read the journal's policy and the authors of this manuscript have the following competing interests: [insert competing interests here]*<br><br>* typeset	
**Ethics Statement**<br><br>Enter an ethics statement for this submission. This statement is required if the study involved:<br><br>• Human participants<br>• Human specimens or tissue<br>• Vertebrate animals or cephalopods<br>• Vertebrate embryos or tissues<br>• Field research<br><br>Write "N/A" if the submission does not require an ethics statement.<br><br>General guidance is provided below. Consult the submission guidelines for detailed instructions. **Make sure that all information entered here is included in the Methods section of the manuscript.**	N/A

Powered by Editorial Manager® and ProduXion Manager® from Aries Systems Corporation

**Format for specific study types**<br><br>**Human Subject Research (involving human participants and/or tissue)**<br>• Give the name of the institutional review board or ethics committee that approved the study<br>• Include the approval number and/or a statement indicating approval of this research<br>• Indicate the form of consent obtained (written/oral) or the reason that consent was not obtained (e.g. the data were analyzed anonymously)<br><br>**Animal Research (involving vertebrate animals, embryos or tissues)**<br>• Provide the name of the Institutional Animal Care and Use Committee (IACUC) or other relevant ethics board that reviewed the study protocol, and indicate whether they approved this research or granted a formal waiver of ethical approval<br>• Include an approval number if one was obtained<br>• If the study involved *non-human primates*, add *additional details* about animal welfare and steps taken to ameliorate suffering<br>• If anesthesia, euthanasia, or any kind of animal sacrifice is part of the study, include briefly which substances and/or methods were applied<br><br>**Field Research**<br><br>Include the following details if this study involves the collection of plant, animal, or other materials from a natural setting:<br>• Field permit number<br>• Name of the institution or relevant body that granted permission	
**Data Availability**<br><br>Authors are required to make all data underlying the findings described fully available, without restriction, and from the time of publication. PLOS allows rare exceptions to address legal and ethical concerns. See the PLOS Data Policy and FAQ for detailed information.	Yes - all data are fully available without restriction

A Data Availability Statement describing where the data can be found is required at submission. Your answers to this question constitute the Data Availability Statement and **will be published in the article**, if accepted.<br><br>**Important:** Stating 'data available on request from the author' is not sufficient. If your data are only available upon request, select 'No' for the first question and explain your exceptional situation in the text box.<br><br>Do the authors confirm that all data underlying the findings described in their manuscript are fully available without restriction?	
**Describe where the data may be found in full sentences. If you are copying our sample text, replace any instances of XXX with the appropriate details.**<br><br>• If the data are **held or will be held in a public repository**, include URLs, accession numbers or DOIs. If this information will only be available after acceptance, indicate this by ticking the box below. For example: *All XXX files are available from the XXX database (accession number(s) XXX, XXX.*).<br>• If the data are all contained **within the manuscript and/or Supporting Information files**, enter the following: *All relevant data are within the manuscript and its Supporting Information files.*<br>• If neither of these applies but you are able to provide **details of access elsewhere**, with or without limitations, please do so. For example:<br><br>*Data cannot be shared publicly because of [XXX]. Data are available from the XXX Institutional Data Access / Ethics Committee (contact via XXX) for researchers who meet the criteria for access to confidential data.*<br><br>*The data underlying the results presented in the study are available*	This is a simulation-based work. Program codes are available via https://github.com/mmsaito/dist-ibm-covid19 and parameter setting for them are stated in the manuscript. As for a detailed procedure to actually run simulator and interpret the result, please see how_to_run_simulator.docx in this repository.

from (include the name of the third party and contact information or URL).<br>• This text is appropriate if the data are owned by a third party and authors do not have permission to share the data.<br><br>* typeset	
Additional data availability information:	

Powered by Editorial Manager® and ProduXion Manager® from Aries Systems Corporation

Manuscript

Click here to access/download;Manuscript;505-09-13-agent-covid-19-PLOSONE-R1-unmarked.docx

Effects of inbound attendees of a mass gathering event on the COVID-19 epidemic using individual-based simulations

Authors:

Masaya M. Saito[1,*], Kotoe Katayama[2], Akira Naruse[3], Peiying Ruan[4], Michio Murakami[5], Tomoaki Okuda[6], Tetsuo Ysutaka[7], Wataru Naito[8], Masaharu Tsubokura[9], Seiya Imoto[2,10]

Affiliations:

[1]Department of Information Security, Faculty of Information Systems, University of Nagasaki, Siebold, Manabino, Nagayocho, Nishisonogigun, Nagasaki, Japan

[2]Laboratory of Sequence Analysis, Human Genome Center, The Institute of Medical Science, The University of Tokyo, Shirokanedai, Minato-ku, Tokyo, Japan

[3]NVIDA, Santa Clara, CA, USA

[4]NVIDIA AI Technology Center, Tokyo, Japan

[5]Center for Infectious Disease Education and Research (CiDER), Osaka University, Yamadaoka, Suita, Osaka, Japan

[6]Department of Applied Chemistry, Faculty of Science and Technology, Keio University, Hiyoshi, Kohoku, Yokohama, Kanagawa, Japan

[7]Institute for Geo-Resources and Environment, National Institute of Advanced Industrial Science and Technology (AIST), Higashi, Tsukuba, Ibaraki, Japan

[8]Research Institute of Science for Safety and Sustainability, National Institute of Advanced Industrial Science and Technology (AIST), Onogawa, Tsukuba, Ibaraki, Japan

[9]Department of Radiation Health Management, Fukushima Medical University School of Medicine, Hikarigaoka, Fukushima, Fukushima, Japan

[10]Division of Health Medical Intelligence, Human Genome Center, The Institute of Medical Science, The University of Tokyo, Shirokanedai, Minato-ku, Tokyo, Japan

*Corresponding author

E-mail: saitohm@sun.ac.jp

Abstract:

Given that mass gathering events involve heterogeneous and time-varying contact between residents and visitors, we sought to identify possible measures to prevent the potential acceleration of the outbreak of an emerging infectious disease induced by such events. An individual-based simulator was built based on a description of the reproduction rate among people infected with the infectious disease in a hypothetical city. Three different scenarios were assessed using our simulator, in which controls aimed at reduced contact were assumed to be carried out only in the main event venue or at subsequent additional events, or in which behavior restrictions were carried out among the visitors to the main event. The simulation results indicated

that the increase in the number of patients with COVID-19 could possibly be suppressed to a level equivalent to that if the event were not being held so long as the prevalence among visitors was only slightly higher than that among domestic residents and strict requirements were applied to the activities of visitors.

1. Introduction

Mass gathering events, such as the Olympic games, world expositions, and music festivals, involve large-scale mixing of people from different regions for extended durations (e.g., durations of a few days to several months). Motivated by the Tokyo 2020 Olympic Games, which were eventually held at the end of July 2021 amidst the coronavirus disease 2019 (COVID-19) pandemic, we aimed to understand the degree to which the reproduction rate can be accelerated by such heterogeneous mixing or be suppressed by behavioral changes from a dynamical perspective. Although an environmental exposure model can quantitatively provide infection risks for various situations [1,2] and be applied to mass gathering events (Murakami, 2020), tracking potential infection events using a mechanistic model could be more effective for devising policies and allowing the general population to understand how such interventions work. For such a mechanistic description, an individual-based (or agent-based) model is known to be useful [3].

The application of agent-based models can be traced back to problems involving the containment of a potential pandemic caused by the novel influenza A virus subtype H5N1 in southeast Asia [4,5]. Ferguson et al. [14] also adjusted their framework to the COVID-19 epidemic in the UK and US in the early stage when non-pharmaceutical interventions were the only available measures and vaccines had yet to be developed. Their simulation results suggested that flattening the epidemic curve was not sufficient to avoid a surge of patients exceeding the capacity of hospitals, and thus, extensive non-pharmaceutical interventions combining home quarantine and social distancing should be administrated while closely monitoring their impacts on the economy and social activities, to reduce the effective reproduction number (R_t) below unity. Referring to mobility and census data, Aleta et al. [6] constructed a detailed agent-based model of transmission in the Boston metropolitan area, and found that social distancing followed by testing and contact tracing could keep the number of cases below the limit of medical capacity. An individual-based model is considered more advantageous than a compartmental one (e.g., the susceptible, infected, and removed or recovered SIR-like model) when an infectious agent spreads among different communities, as it is highly stochastic in the early stage of the epidemic and keen to provide a correct description of the transmission dynamics. The most of infected people, in general, lose their infectiousness without yielding the successor generations, which are attributable to quite small portion of the predecessors, the so-called super spreaders. The super spreaders are visible in a small outbreak; in MERS 2015 nosocomial outbreak, for example, these were identified as the starting nodes of transmission chain [7] and a single component having a significantly higher reproduction number than the rest of components in the next generation matrix [8]. There possibly exist common risk factors of contracting SARS-CoV-2 behind the superspreading, and a multinational cross-sectional study [9] found as significant factors, history of being infected to SARS-CoV or MERS-CoV, habit of hugging when greeting, and smoking. Son et al. [Son WS, RISEWIDs Team. Individual-based simulation model for COVID-19 transmission in Daegu, Korea. Epidemiology and

Health 2020; 42: e2929942.] successfully predicted the number of cases that would arise in Daegu, South Korea by using an agent-simulation model, where the majority of reported cases at that time (end of March 2020) were tied to a single church and the transition from the church to other communities was a key factor in the prediction. Religion-related mass gatherings were held also in other countries during a pandemic wave growing; the Kumbh Mela celebration lasted for one month since April 1, 2021 in India [11] and Songkran Buddhist new year celebration from April 12 to 15, 2021 in Thailand [12].

Individual-based simulation has traditionally been used to target problems in epidemics involving the mixing of heterogenous populations and time-varying contact rates. Assessing the influence of a mass gathering event on an epidemic falls into this type of problem, where infectious cases from outside may have a non-small effect on the R_t even among local residents. In particular, the total number of visitors and the prevalence of infection among them, which may be comparable to the residents in the region, are key factors in such predictions. For this reason, we built an individual- (or agent)-based model with inflow and assessed how the growth of new cases could be controlled by different interventions under different prevalence rates among visitors.

2. Methods

2.1 Individual-based model

A mass gathering event yields additional gathering occasions, called secondary gatherings, that involve local and outside populations. For an appropriate description of transmission dynamics, the import of infected people between two types of temporarily formed populations should be considered. For this reason, we employed an individual-based description of the epidemic rather than a compartmental one such as the SIR model.

Specifically, we constructed an individual-based model by extending our study of the 2009 influenza pandemic [13] to deal with main and secondary gathering events. A simulation implementing the baseline model describes the activity of residents in a hypothetical city consisting of five towns that are tightly connected via commuter train, each of which being a collection of households, companies, schools, and shopping centers. Temporal population in these areas are formed by role-based activity of agents, classified into students, office workers, housekeepers, and visitors from outside the city. The state of an individual with respect to the disease is classified in the same manner as the SEIR model, that is, susceptible (S), infected but not yet infectious (E), infected and infectious (I), and immune (R). In each local place, susceptible agents suffer infection risk P during the small time period $\Delta t = 1\text{min}$:

$$P = R_t \cdot \frac{\Delta t}{T_{\text{inf}}} \cdot \frac{I}{N} \ ,$$

where N and I are the (temporal) numbers of total and infectious visitors, respectively, R_t is the effective reproduction number in this place, and $T_{\text{inf}} = 4\text{day}$ is the mean infectious period. This city is a simplified model of central Tokyo; a schematic illustration of this city is shown in Figure 1, and the numbers of entities are listed in Table 1. In total, about 1 million residents live in the city and have the chance to visit the main event, which is set to have 50,000 attendees. The timing of attendance is random.

For meanwhile, put the sentences to define the simulator at the end of the paragraph:
The main mass gathering event consists of sub-venues, each of which randomly selected residents are scheduled to attend, and attendees in different sub-events are mutually segregated.

Table 1. Populations and number of places in the simulation.

	**Town 1**	**Town 2**	**Town 3**	**Town 4**	**Town 5**
Population	571,641	176,866	138,684	314,861	44,680
#Shops	100	100	100	100	100
#Parks	2	2	2	2	2
#Schools	70	20	12	29	8
#Offices	100	100	100	2,000	2,000

Figure 1. Schematic illustration of the simulated city.

The simulated city is designed so that a set of places have only a threshold-level reproduction number, but an activity of visitors higher than residents could alter the tread in the number of new cases. Below, we briefly introduce the assumptions on the situation in the simulated city and the reasoning behind them. It is unlikely that the epidemic in concern is highly reproducible when a mass gathering event is allowable. For this reason, the transmissibility parameters in populations in the simulated city have been configured so that the R_t of the entire city is nearly equal to unity and the number of daily cases is kept almost constant until the event. The game venue is also not expected to greatly modify the trend in total number of new cases. However, a non-small number of attendees would be expected to visit some other location after the event is over in order to meet with or socialize with one another, thereby creating many secondary gathering events. Some of these gatherings cause potentially the so-called super-spreading. Accordingly, game watchers in simulation are scheduled probabilistically to attend additional gathering with a variety infection risk there. Specifically, this calibration was carried out in a heuristic manner because unlike compartmental models, this critical condition is not described in a simple formula. We further assume that inbound attendees come to the main event multiple times and always join an additional event after the main one, considering that such travelers would necessarily eat out, in contrast to local attendees. The abovementioned assumptions are reflected in the parameter setting shown in Table 2, where the infection risk is quantified in terms of the R_t. In particular, a heavy tailed distribution of Rt at secondary gathering places is introduced to describe potential superspreading events. In households, schools, and corporations, the risk is small, unlike the 2009 swine flu pandemic. Therefore, the R_t values are set to 1. The attributed R_t value, 1.05, to places other than those for the main and sub-events was chosen heuristically to establish a sustained (non-growing) epidemic. The

proportion of the main event attendees who are dropped (e.g., to meet their friends) is provided by a probabilistic distribution.

Table 2. Parameters defining the activity of people in the simulation.

Reproduction number ($\mathbf{R}_t$)			
	Main events	25 (baseline) or 1 (controlled)	
	Secondary events (uncontrolled)*	17-24 (90%), 240 (10%)	
	Secondary events (controlled)*		
	Other sites	1	
		Domestic	Inbound
Prevalence		0.01%	≦1% (secenario-dependent)
Per capita attendance times		1	1-3
Probability of a seconcary event*		0-0.1 (50%), 0.1-0.9 (40%), 0.9-1 (10%)	1

2.2 Scenarios

To examine the possibility of suppressing the additional impetus to the epidemic brought by visitors, we carried out simulations with the following three scenarios:

1. **StadiumOnly:** The control is administered only in the main event venue. In the simulation, the control is implemented by a reduction in the R_t from 25 to 1 (see Table 2).
2. **Extra:** In addition to StadiumOnly, secondary events are assumed to be controlled. This is implemented by reducing the proportion of highly risky places (R_t = 240) for secondary events from 10% to 1%.
3. **WithoutDrop:** In addition to StadiumOnly, most of the attendees (specifically 90% in the simulation) are assumed to go directly home in accordance with a corresponding governmental campaign. The risk distribution in secondary events is the same as that in the StadiumOnly scenario.

The abovementioned values are arbitrary, but reflect the following assessment. First, in the simulations, we set the latent period to 3.5 days and the infectious period to 3 days, as average. We here assign rather shorter values, considering these periods are exponentially distributed in our model while the reality is close to a gamma or Weibull distribution [14]. Second, Murakami [15] assessed the infection risk in the Opening Ceremonies of the Tokyo 2020 Olympic Games as follows. An infectious attendee would yield about 1.5 or 0.01 new cases in uncontrolled and controlled settings, respectively, during a 5-hour-long ceremony, which is translated into the $\mathbf{R}_t$ by:

$$\mathbf{R}_t = (3\text{ days})/(5\ /\ 24\text{ days}) \times (1.5\text{ or }0.01)\ \text{ per capita cases} \approx 22\text{ or }0.1.$$

As for the controlled case, we used the threshold value, R_t = 1, instead for this rather small risk. A similar assessment is available for another event, a 4-hour long soccer game: 0.74 or 0.022 cases are reproduced by

one infectious case, respectively. A higher risk at some of the secondary gatherings is based on a survey targeting a large indoor convention [16]. That study showed that respondents testing positive were more likely to have attended pubs, karaoke bars, or nightclubs. Using their reported values, attendees to these additional activities are at roughly three times higher risk of infection. Further assuming that this tripled risk is experienced within 1.5 hours, we take $\mathrm{R}_t = 24 \times 5/1.5 \times 3 = 240$ to be the worst case scenario at additional meetings.

3. Results

Whether main mass gathering events accelerate epidemics depends on the baseline setting, the prevalence rate among visitors, and possible counter-measures to help prevent close contact. Figure 2 summarizes the outcome under each setting as an epidemic curve. As a baseline, the transmissibility parameters in respective small populations in the simulated city were configured so that the R_t of the entire city remained nearly equal to unity until the event opened. As was configured, the number of new cases remained almost constant until the main event opened on day 14, and then tended to increase with a different slope until the event closed on day 24.

With the measure against only the event venue (scenario **StadiumOnly**) and zero infectious visitors, the new cases increased with a slope of $R_t = 1.08$, as shown at the top left of Figure 2. This increasing trend was sustained when the prevalence of infection among the visitors was mildly high, $p_{\mathrm{inf,vis}} = 0.1\%$, but rapidly increased to $R_t = 1.33$ when the prevalence of infection was extremely high, at $p\_(\mathrm{inf,vis}) = 1\%$. These results suggest that cases imported from the outside would have only a minor impact if $p_{\mathrm{inf,vis}} < 0.1\%$.

Regarding secondary events under scenario **Extra**, it may be possible to erase the uptrend attributable to the main event when the visitors are fully noninfectious. Infectious visitors may reinstate an uptrend, but our findings suggest that it would be rather weak as long as the prevalence was mild: $R_t = 1.04$ for $p\_(\mathrm{inf,vis}) = 0.1\%$. A recommendation to visitors to return directly home (scenario **WithoutDrop**) would have a similar effect. A constant trend would be sustained after the start of the event start if no infectious visitors came in, or a shift to a weak uptrend if their prevalence rate was <0.1%.

So far, we have interpreted the impacts of infectious visitors and controls, viewing only the mean epidemic curve trajectories. However, these curves are highly stochastic, as shown in the gray shading in each panel of Figure 2, and the slope of the trend involves a greater uncertainty, as shown in Figure 3. Figure 4 shows the R_t with an uncertainty for all scenarios as a function of $p_{\mathrm{inf,vis}}$. The strongest measure of **WithoutDrop** suppresses the increase in the R_t based on the prevalence rate of visitors. On average, $p\_(\mathrm{inf,vis}) \leq 0.1\%$ keeps the R_t almost at unity under this measure.

Figure 2. Simulated epidemic curves for different assumptions on the prevalence rate among visitors and possible measures. Thick lines indicate the mean (blue) and median (red) trajectories, while thin lines correspond to respective quantiles (25th, 75th, 80th, 85th, 90th, and 95th percentiles). The main mass gathering event runs from day 14 through 23 and is bounded by vertical lines.

Figure 3. The effective reproduction number (R_t) in the entire city during the main event in each scenario. The uncertainty in each setting is evaluated from 1,000 runs with a different random number sequence.

Figure 4. Distributions of cumulative cases during the event corresponding to Figure 2. The uncertainty is based on 1,000 simulation runs.

4. Discussion and Conclusion

In this study, we demonstrated how the potential growth of an epidemic can be successfully or unsuccessfully suppressed in a mass gathering event involving a comparable number of local and outside visitors depending on the strength of the intervention and the prevalence rate among visitors. As a general remark, the growth rate potentially enhanced by hosting a mass gathering can be suppressed to a level identical or slightly higher than that of the baseline if the places for gathering after the main event are well controlled and the prevalence rate among visitors is mild relative to that among local residents.

The controls discussed in this work are voluntary-basis and hence the degree people are willing to cooperate limits the effectiveness. A massive superspreading along with Kumpha Mela [11] religious could be numbered as a case that such a cooperation is poorly established, and many attendees reportedly did not follow to the requests of mask wearing and social distancing. Though we are not sure how to measure directly such a willingness via investigation, we can refer some articles which help future discussion. People yielding higher score in willingness to cooperate may not show a greater acceptance of behavioral regulations [17]. The cultural difference affects on which of individual-basis or law-enforced measures are preferable. A comparative study of three countries [18] shows Chinese participants are more acceptable to enforced measures than Japan and US, while US and Chinese more accept individual-level measures than does Japanese. Another investigation [19] supports that Japanese people are reluctant to enforced restrictions in a greater degree than the other participating countries. As for mask wearing, one of self-motivated measures, there are studies over 50 US states and over the world that support people in collectivistic communities more frequently wear masks than in individual ones [20]. Also, it is possible to build a cooperative relationship among supports fan, academic experts, and event organizers: as high as about 98% of chant cheering participants wore masks in games held by Japan Football leagues [21].

Reflecting the lack of qualitatively interpretable data for shops, restaurants, and other kinds of possible meeting places, we carried out simulations with scenario-based settings to assess risk in these places. Below, we discuss the availability of data from the parameters of our simulation. The Japanese government enacted a strict quarantine at airports during the pandemic. The data collected from the quarantine [22,23]

could be expected to aid the estimation of prevalence rates among visitors, thereby making quarantine more effective in combination with testing before boarding and after arrival [24].

In addition to the prevalence obtained along with the quarantine, the prevalence rate among the participants of the Tokyo 2020 Olympic Games is available [25]. Maintaining a sufficient level of control at secondary gathering places is more difficult than the main event venue. The Tokyo Metropolitan Government issued an "Infection Prevention Thorough Declaration Sticker" to shops, restaurants, and other meeting places to establish a certain level of risk reduction and provide information on well-sanitized places to local residents [26]. Hence, the ratio of safe places to 'risky places' (10% or 1% being assumed in the present study) may be determined via data. However, the requirements for this are mainly qualitative, and it is nontrivial to translate these data into the risk of infection computationally interpretable.

Observational facts that contribute to connecting the emission of droplets to the risk of infection have accumulated since the beginning of the COVID-19 pandemic and the abovementioned difficulties may be overcome by plugging these data into simulations. While some of this knowledge has yet to be fully authorized, a number of studies have already been published. Ando et al. [27] assessed infection risk at a restaurant using a dedicated hydrodynamical model on the Fugaku supercomputer. The effects of air ventilation in the venue have been monitored based on CO_2 concentrations during a football game [28], and droplet emissions have been measured during normal speech [29], coughing [30], and singing [31]. In addition, a COVID-19 outbreak event suspected to be induced by air conditioning in a restaurant has been reported [32]. We expect that a reliable set of infection risk parameters will become available based on the outcomes of empirical studies interpreted inside hydrodynamical droplet simulations, and that individual-based models will contribute to improved risk assessments against new variants of severe acute respiratory syndrome coronavirus 2 and future pandemics caused by other infectious diseases.

References

1. Jones RM. Relative contributions of transmission routes for COVID-19 among healthcare personnel providing patient care. J. Occup. Environ. Hyg. 2020;17: 408–415.
2. Nicas M, Sun G, 2006. An integrated model of infection risk in a health-care environment. Risk Anal 2006; 26: 1085–1096.
3. Riley S. Large-Scale Spatial-Transmission Models of Infectious Disease. Science 2007;316: 1298-1301. doi: 10.1126/science.1134695.
4. Ferguson NM et al. Strategies for containing an emerging influenza pandemic in Southeast Asia. Nature 2005;437: 209-14. doi: 10.1038/nature04795.
5. Longini IM, Jr. et al. Containing Pandemic Influenza at the Source. Science 2005; 309: 1083-87.
6. Aleta A, Martin-Corral D, Pastore y Piontti A, Ajelli M, Litvinova M, Chinazzi M, Dean NE, Halloran ME, Longini IM Jr, Merler S, Pentland A, Vespignani A, Moro E, Moreno Y. Modelling the impact of testing, contact tracing and household quarantine on second waves of COVID-19. Nature Human Behaviour 2020; 4: 964-971.
7. Cowling BJ, Park M, Fang VJ, Wu P, Leung GM, Wu JT. Preliminary epidemiological assessment of

MERS-CoV outbreak in South Korea, May to June 2015. Euro Surveill. 2015;20(25):pii=21163. Available online: http://www.eurosurveillance.org/ViewArticle.aspx?ArticleId=21163

8. Nishiura H, Endo A, Saito MM, Kinoshita R, Ueno R, Nakaoka S, Miyamatsu Y et al. Identifying determinants of heterogeneous transmission dynamics of the Middle East respiratory syndrome (MERS) outbreak in the Republic of Korea, 2015: a retrospective epidemiological analysis. BMJ Open. 2016;6:e009936. doi: 10.1136/bmjopen-2015-009936
9. Mai NL, Alhady STM, Nguyen TMD, Le VT, Qarawi A, Venkatesh U, et al. Evaluation of risk factors associated with SARS-CoV-2 transmission, Current Medical Research and Opinion. Current Medical Research and Opinion. 2022; 38(12), 2021-2028. doi: 10.1080/03007995.2022.2125258
10. Son WS, RISEWIDs Team. Individual-based simulation model for COVID-19 transmission in Daegu, Korea. Epidemiology and Health 2020; 42: e2929942.
11. Rocha ICN, Pelayo MGA, Rackimuthu S. Kumbh Mela Religious Gathering as a Massive Superspreading Event: Potential Culprit for the Exponential Surge of COVID-19 Cases in India. Am. J. Trop. Med. Hyg. 2021;105(4), 868-871. doi: 10.4269/ajtmh.21-0601
12. Rocha ICN, Pelayo MGA, Sammatid C. Traveling and Celebrating During Songkran as Super Spreading Events: A Potential Triggering Factor of the Surge of COVID-19 Cases in Thailand. Int J Travel Med Glob Health. 2021;9(4):196-197. doi 10.34172/ijtmgh.2021.33
13. Saito MM et al. Enhancement of collective immunity in Tokyo Metropolitan area by selective vaccination against an emerging influenza pandemic. PLOS ONE 2013; 8: e72866. doi: 10.1371/journal.pone.0072866.
14. Ferguson N et al. Report 9: Impact of non-pharmaceutical interventions (NPIs) to reduce COVID19 mortality and healthcare demand. 2020. doi: 10.25561/77482.
15. Murakami et al. COVID-19 risk assessment at the opening ceremony of the Tokyo 2020 Olympic Games. Microbial Risk Analysis 2020;19:100162. doi: 10.1016/j.mran.2021.100162.
16. Sami S et al. 2022. Investigation of SARS-CoV-2 Transmission associated with a large indoor convention — New York City, November–December 2021. Morbidity and Mortality Weekly Report 2022; 71(7): 243-248. doi: http://dx.doi.org/10.15585/mmwr.mm7107a4
17. Zhu N, Smetana JG, Chang L. Acceptance of Society-Level and Individual-Level Preventive Measures during the COVID-19 Pandemic among College Students in Three Societies. Journal of Cross-Cultural Psychology 2021; 52(7): 606–621. https://journals.sagepub.com/doi/10.1177/0022022121995971?icid=int.sj-full-text.similar-articles.5
18. Romano A, Spadaro G, Balliet D, Joireman J, van Lissa C, Jin S, Agostini M, Bélanger JJ, Gützkow B, Kreienkamp J, Leander NP. Cooperation and Trust Across Societies During the COVID-19 Pandemic. Journal of Cross-Cultural Psychology 2021; 52(7):622–642. https://journals.sagepub.com/doi/full/10.1177/0022022120988913
19. Murakami M, Hiraishi K, Yamagata M, Nakanishi D, Ortolani A, Mifune N, Li Y, Miura A. 2023. Differences in and associations between belief in just deserts and human rights restrictions over a 3-year period in five countries during the COVID-19 pandemic. PeerJ 2023; 11:e16147

http://doi.org/10.7717/peerj.16147

20. Lu JG, Jin P, English AS. Collectivism predicts mask use during COVID-19. PNAS 2021;118(23). https://doi.org/10.1073/pnas.2021793118
21. Yasutaka T, Onishi M, Naito W, Bando Y, Okuda T. Maximum proportion of masks worn: Collaborative efforts in Japanese professional football. Journal of Infection and Public Health 2023. https://doi.org/10.1016/j.jiph.2023.03.009
22. Ministry of Health, Labour and Welfare (MHLW). Press release of COVID (domestic new cases and quarantine at air- and seaports; in Japanese). 2021. Available from: https://www.mhlw.go.jp/stf/seisakunitsuite/bunya/0000121431_00254.html
23. Tokyo Metropolitan Government. Tokyo Metropolitan Government COVID-19 monitoring meeting minutes (in Japanese). 2020. Available from: URL: https://www.bousai.metro.tokyo.lg.jp/taisaku/saigai/1009676/index.html
24. Kamo M, Murakami M, Imoto S. Effects of test timing and isolation length to reduce the risk of COVID-19 infection associated with airplane travel, as determined by infectious disease dynamics modeling. Microbial Risk Analysis 2022;20: 1-8. doi: 10.1016/j.mran.2021.100199.
25. Tokyo Metropolitan Government. The values Tokyo metropolitan brought to us (in Japanese). 2021. Available from: https://www.2020games.metro.tokyo.lg.jp/special/1b340dde24650e2db8121738cef945cf.pdf
26. Tokyo Metropolitan Government. Infection Prevention Thorough Declaration Sticker (in Japanese). 2021. Available from: https://web.archive.org/web/20230404213919/https://www.bousai.metro.tokyo.lg.jp/taisaku/torikumi/1008262/1008420/index.html
27. Ando K, Bale R, Li CG, Matsuoka S, Onishi K, Tsubokura M. Digital transformation of droplet/aerosol infection risk assessment realized on "Fugaku" for the fight against COVID-19. to appear on International Journal of High-Performance Computing Application, to appear (preprint is available from arXiv:2110.09769v3).
28. Murakami M, Yasutaka T, Onishi M, Naito W, Shinohara N, Okuda T, Fujii K, Katayama K, Imoto, S. Living with COVID-19: mass gatherings and minimizing risk. QJM: An International Journal of Medicine 2021; 114 (7): 437–439. doi:10.1093/qjmed/hcab163.
29. Asadi S, Wexler AS, Cappa CD, Barreda S, Bouvier NM, Ristenpart WD. Aerosol emission and superemission during human speech increase with voice loudness. Scientific Reports 2019; 9:2348. doi:10.1038/s41598-019-38808-z
30. Milton DK, Fabian MP, Cowling BJ, Grantham ML, McDevitt JJ. Influenza Virus Aerosols in Human Exhaled Breath: Particle Size, Culturability, and Effect of Surgical Masks. PLoS Pathog 2013;9(3): e1003205. doi:10.1371/journal.ppat.
31. Alsveda M, Matamisb A, Bohlinc R, Richterb M, Bengtssonb PE, Fraenkeld CJ, Medstrande P, Londahl J. Exhaled respiratory particles during singing and talking. Aerosol Scicence and Technology

2020;54(11): 1245–1248. doi: 10.1080/02786826.2020.1812502.

32. Lu J, Gu J, Li K, Xu C, Su W, Lai Z, Zhou D, Yu C, Xu B, Yang Z. COVID-19 Outbreak Associated with Air Conditioning in Restaurant, Guangzhou, China, 2020. Emerging Infectious Diseases 2020; 26(7): 1628-31.

Click here to access/download;Figure;Fig1_schematic_illust.png

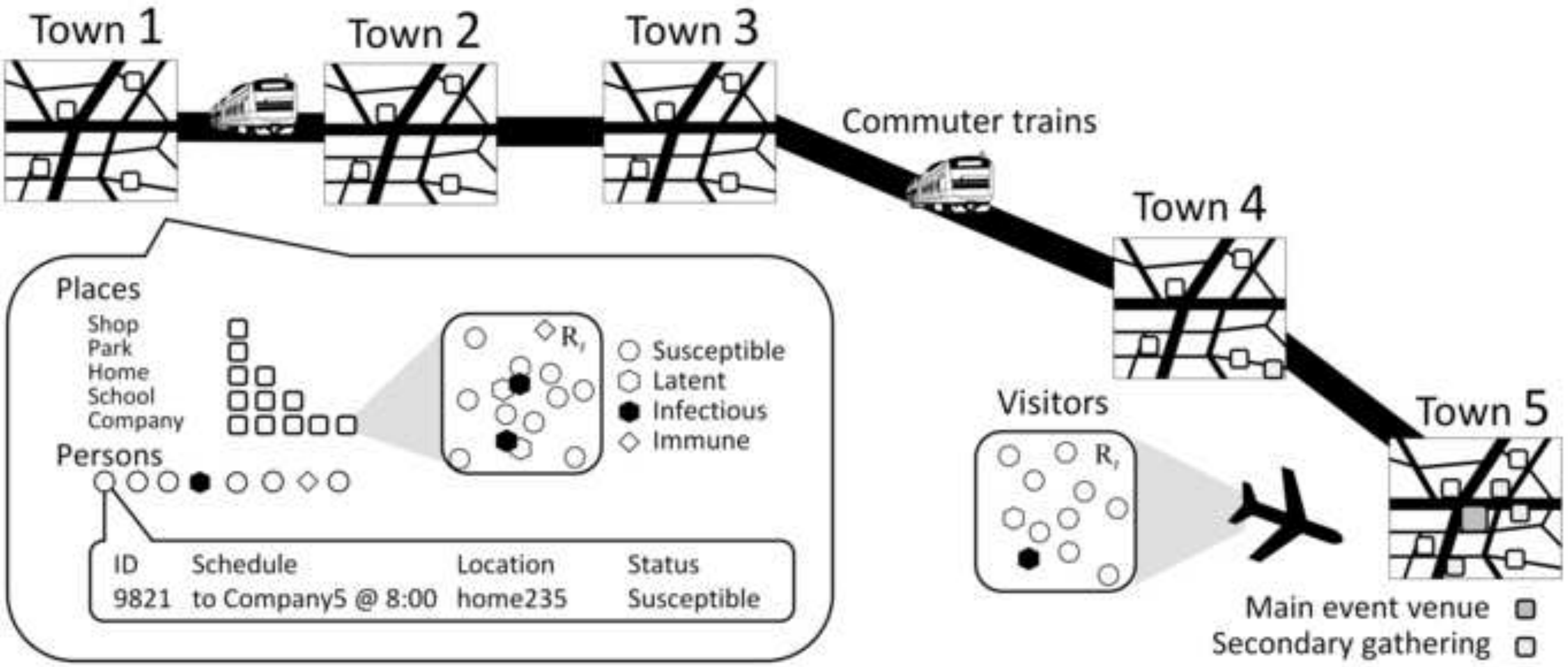

Figure 2

Click here to access/download;Figure;Fig2_matrix-pop-inf-town#1-edit2.png

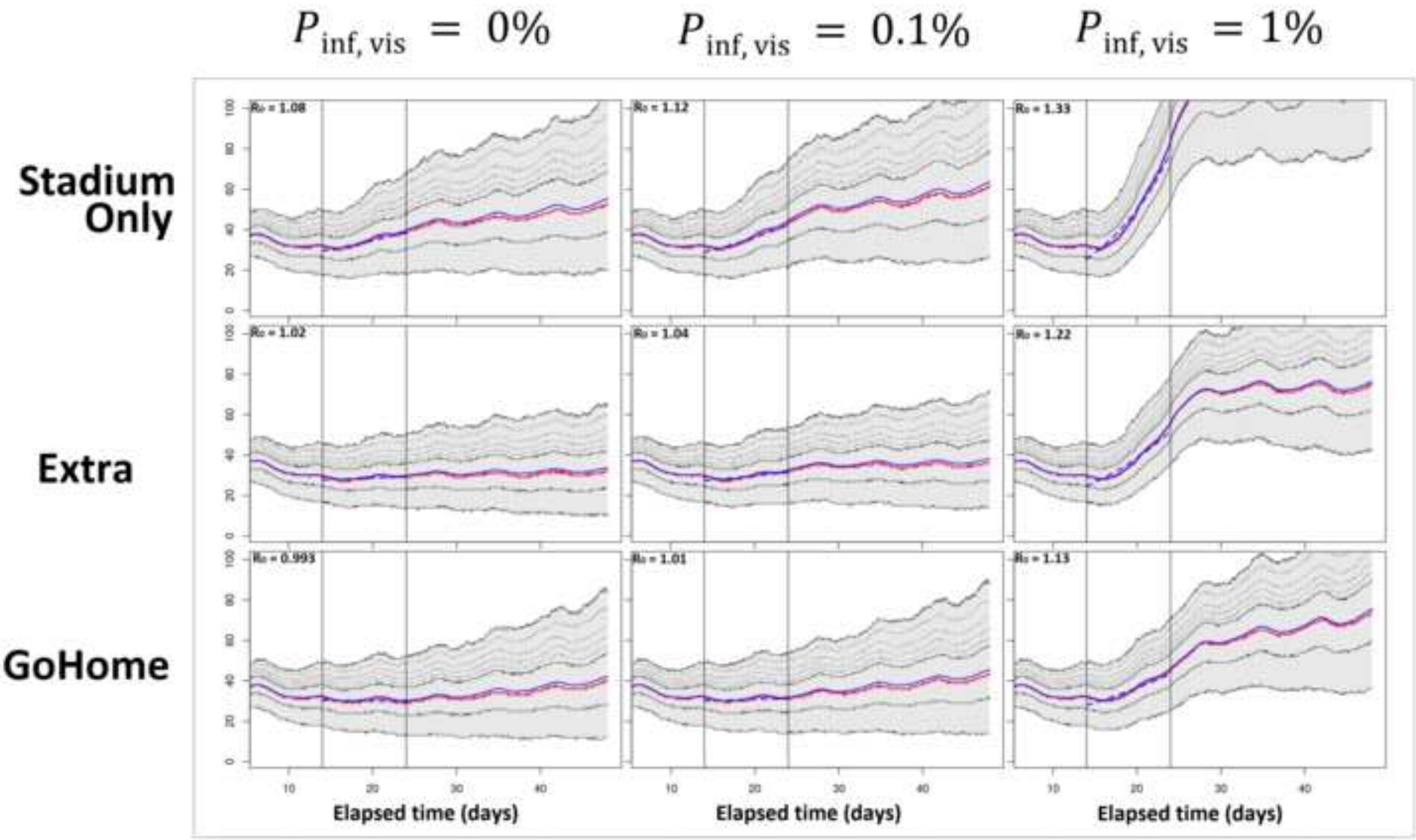

Figure 3

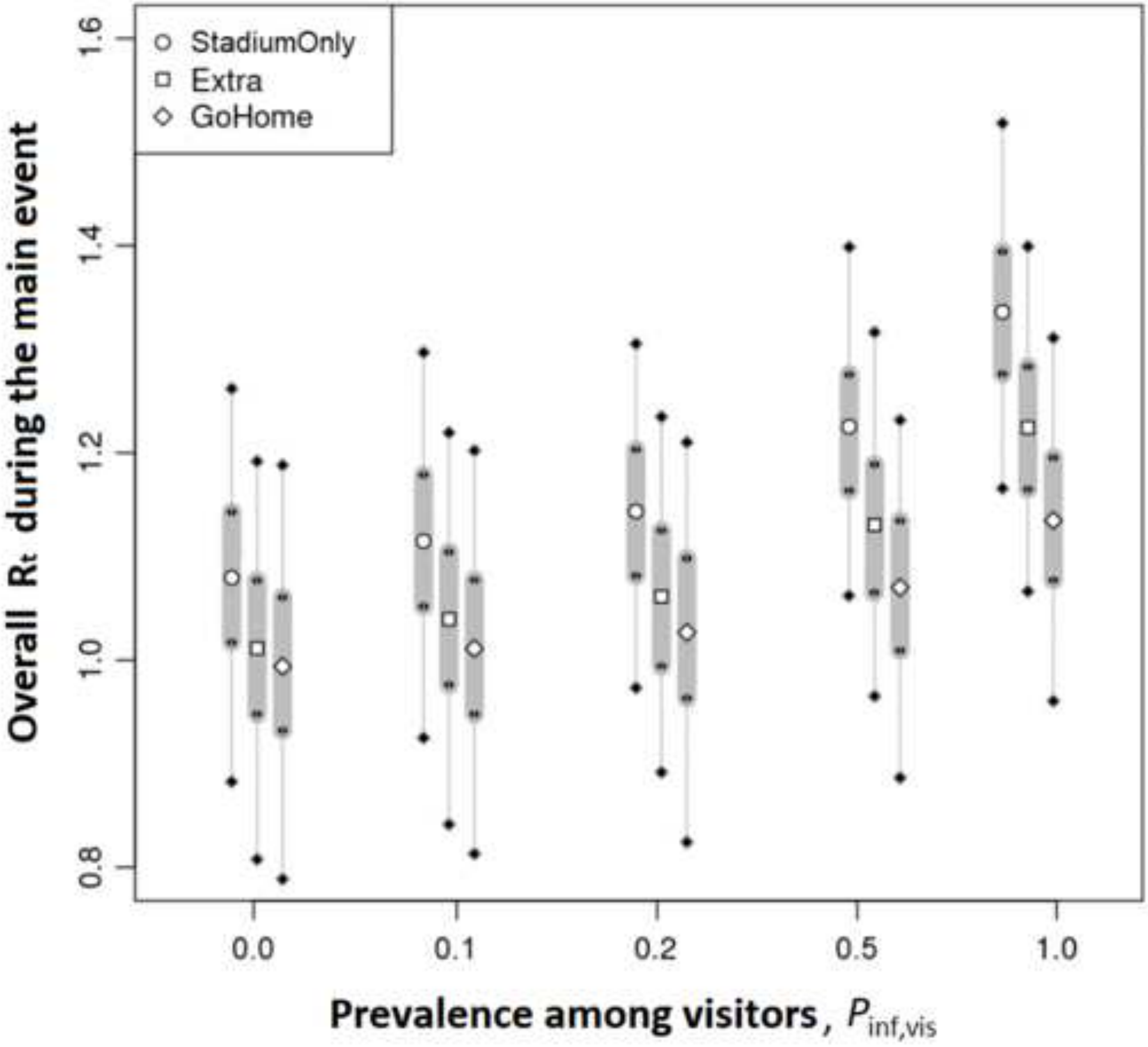
StadiumOnly
Extra
GoHome
1.6
1.4
1.2
1.0
0.8
Overall Rt during the main event
0.0
0.1
0.2
0.5
1.0
Prevalence among visitors, P inf,vis

Figure 4 Click here to access/download;Figure;Fig4_barplot-town1.png

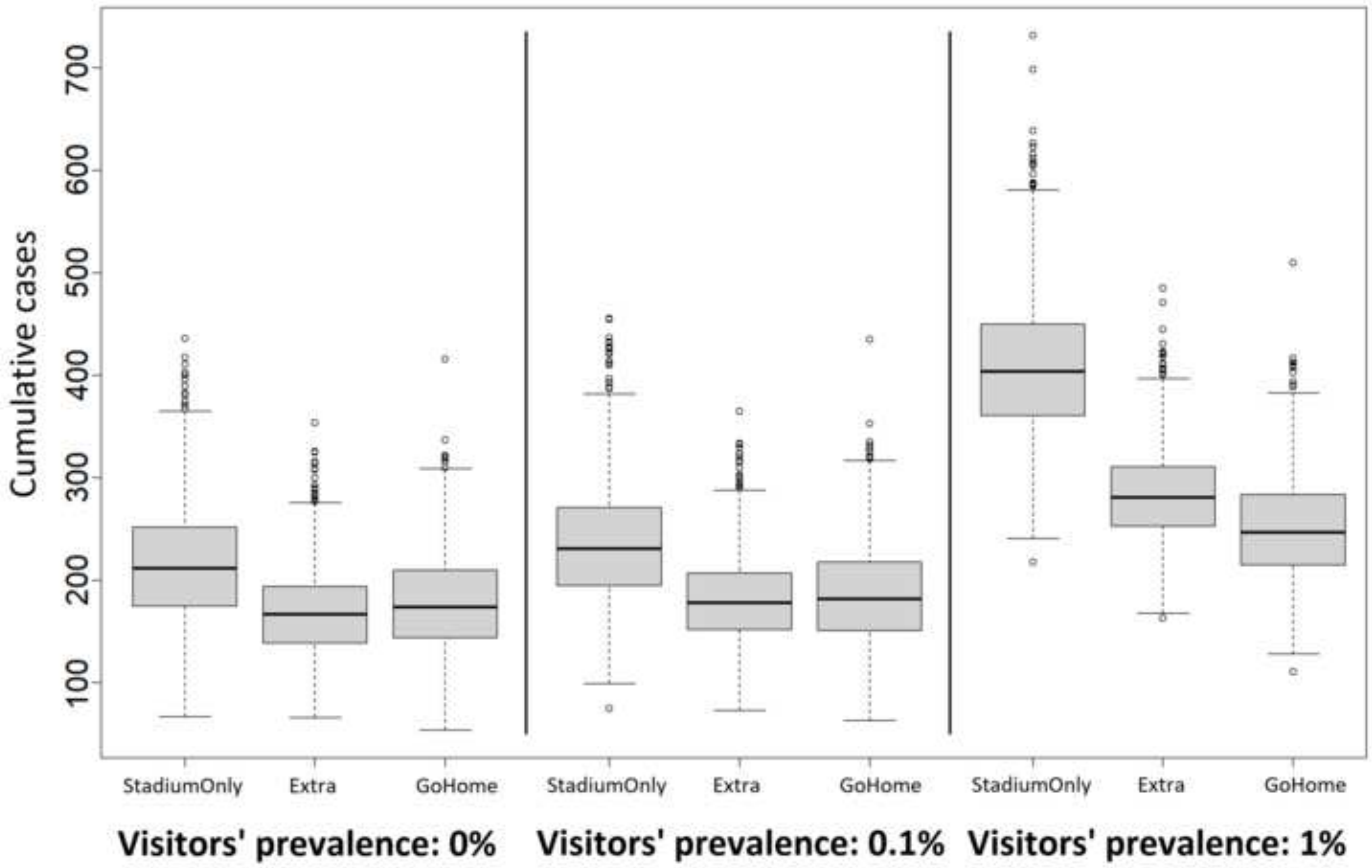

How to run the simulator

Click here to access/download
Supporting Information
how_to_run_simulator.docx

Effects of inbound attendees of a mass gathering event on the COVID-19 epidemic using individual-based simulations

Authors:

Masaya M. Saito[1,*], Kotoe Katayama[2], Akira Naruse[3], Peiying Ruan[4], Michio Murakami[5], Tomoaki Okuda[6], Tetsuo Ysutaka[7], Wataru Naito[8], Masaharu Tsubokura[9], Seiya Imoto[2,10]

Affiliations:

[1]Department of Information Security, Faculty of Information Systems, University of Nagasaki, Siebold, ~~1-1-1,~~ Manabino, Nagayocho, Nishisonogigun, Nagasaki ~~851-2195,~~, Japan

[2]Laboratory of Sequence Analysis, Human Genome Center, The Institute of Medical Science, The University of Tokyo, ~~4-6-1~~ Shirokanedai, Minato-ku, Tokyo ~~108-8639~~, Japan

[3]NVIDA, Santa Clara, CA, USA

[4]NVIDIA AI Technology Center, Tokyo, Japan

[5]Center for Infectious Disease Education and Research (CiDER), Osaka University, ~~2-8~~ Yamadaoka, Suita, Osaka ~~565-0871~~, Japan

[6]Department of Applied Chemistry, Faculty of Science and Technology, Keio University, ~~3-14-1,~~ Hiyoshi, Kohoku, Yokohama, Kanagawa ~~223-8522~~, Japan

[7]Institute for Geo-Resources and Environment, National Institute of Advanced Industrial Science and Technology (AIST), ~~1-1-1~~ Higashi, Tsukuba, Ibaraki ~~305-8567~~, Japan

[8]Research Institute of Science for Safety and Sustainability, National Institute of Advanced Industrial Science and Technology (AIST), ~~16-1,~~ Onogawa, Tsukuba, Ibaraki ~~305-8569~~, Japan

[9]Department of Radiation Health Management, Fukushima Medical University School of Medicine, ~~1~~ Hikarigaoka, Fukushima, Fukushima ~~960-1295~~, Japan

[10]Division of Health Medical Intelligence, Human Genome Center, The Institute of Medical Science, The University of Tokyo, ~~4-6-1~~ Shirokanedai, Minato-ku, Tokyo ~~108-8639~~, Japan

*~~Address c~~Corresponding author~~to Masaya M. Saito, 1-1-1 Manabino, Nagayo-cho, Nishi-Sonogi-gun, Nagasaki 851-2195~~

E-mail: saitohm@sun.ac.jp

Abstract:

Given that mass gathering events involve heterogeneous and time-varying contact between residents and visitors, we sought to identify possible measures to prevent the potential acceleration of the outbreak of an emerging infectious disease induced by such events. An individual-based simulator was built based on a description of the reproduction rate among people infected with the infectious disease in a hypothetical city. Three different scenarios were assessed using our simulator, in which controls aimed at reduced contact were

Formatted: Highlight

Commented [A1]: Adjusted to the style guid

Formatted: Highlight

assumed to be carried out only in the main event venue or at subsequent additional events, or in which behavior restrictions were carried out among the visitors to the main event. The simulation results indicated that the increase in the number of patients with COVID-19 could possibly be suppressed to a level equivalent to that if the event were not being held so long as the prevalence among visitors was only slightly higher than that among domestic residents and strict requirements were applied to the activities of visitors.

1. Introduction

Mass gathering events, such as the Olympic games, world expositions, and music festivals, involve large-scale mixing of people from different regions for extended durations (e.g., durations of a few days to several months). Motivated by the Tokyo 2020 Olympic Games, which were eventually held at the end of July 2021 amidst the coronavirus disease 2019 (COVID-19) pandemic, we aimed to understand the degree to which the reproduction rate can be accelerated by such heterogeneous mixing or be suppressed by behavioral changes from a dynamical perspective. Although an environmental exposure model can quantitatively provide infection risks for various situations [1,2] and be applied to mass gathering events (Murakami, 2020), tracking potential infection events using a mechanistic model could be more effective for devising policies and allowing the general population to understand how such interventions work. For such a mechanistic description, an individual-based (or agent-based) model is known to be useful [3].

The application of agent-based models can be traced back to problems involving the containment of a potential pandemic caused by the novel influenza A virus subtype H5N1 in southeast Asia [4,5]. Ferguson et al. [14] also adjusted their framework to the COVID-19 epidemic in the UK and US in the early stage when non-pharmaceutical interventions were the only available measures and vaccines had yet to be developed. Their simulation results suggested that flattening the epidemic curve was not sufficient to avoid a surge of patients exceeding the capacity of hospitals, and thus, extensive non-pharmaceutical interventions combining home quarantine and social distancing should be administrated while closely monitoring their impacts on the economy and social activities, to reduce the effective reproduction number (R_t) below unity. Referring to mobility and census data, Aleta et al. [6] constructed a detailed agent-based model of transmission in the Boston metropolitan area, and found that social distancing followed by testing and contact tracing could keep the number of cases below the limit of medical capacity. An individual-based model is considered more advantageous than a compartmental one (e.g., the susceptible, infected, and removed or recovered SIR-like model) when an infectious agent spreads among different communities, as it is highly stochastic in the early stage of the epidemic and keen to provide a correct description of the transmission dynamics. ~~[erase break]{~~

The most of infected people, in general, lose their infectiousness without yielding the successor generations, which are attributable to quite small portion of the predecessors, the so-called super spreaders. The super spreaders are visible in a small outbreak; in MERS 2015 nosocomial outbreak, for example, these were identified as the starting nodes of transmission chain [1~~cowling2015~~] and a single component having a significantly higher reproduction number than the rest of components in the next generation matrix [1

Formatted: Highlight

Formatted: Font: Not Bold, Highlight

Formatted: Font: Not Bold, Highlight

Formatted: Highlight

Formatted: Font: Not Bold, Highlight

Formatted: Font: Not Bold, Highlight

~~nishiura2015~~]. There possibly exist common risk factors of contracting SARS-CoV-2 behind the superspreading, and a multinational cross-sectional study [1~~Reviewers 3-1~~] found as significant factors, history of being infected to SARS-CoV or MERS-CoV, habit of hugging when greeting, and smoking.

~~[Korean case comes here]~~

~~Religion-related mass gatherings were held also in other countries during a pandemic wave growing; the Kumbh Mela celebration lasted for one month since April 1, 2021 in India and Songkran Buddhist new year celebration from April 12 to 15, 2021.~~

~~1~~

Son et al. [~~7~~Son WS, RISEWIDs Team. Individual-based simulation model for COVID-19 transmission in Daegu, Korea. Epidemiology and Health 2020; 42: e2929942.] successfully predicted the number of cases that would arise in Daegu, South Korea by using an agent-simulation model, where the majority of reported cases at that time (end of March 2020) were tied to a single church and the transition from the church to other communities was a key factor in the prediction. ~~[Korean case comes here]~~

Religion-related mass gatherings were held also in other countries during a pandemic wave growing; the Kumbh Mela celebration lasted for one month since April 1, 2021 in India [1~~Reviewer 3-2~~] and Songkran Buddhist new year celebration from April 12 to 15, 2021 in Thailand [1~~Reviewer 3-23~~].

Individual-based simulation has traditionally been used to target problems in epidemics involving the mixing of heterogenous populations and time-varying contact rates. Assessing the influence of a mass gathering event on an epidemic falls into this type of problem, where infectious cases from outside may have a non-small effect on the R_t even among local residents. In particular, the total number of visitors and the prevalence of infection among them, which may be comparable to the residents in the region, are key factors in such predictions. For this reason, we built an individual- (or agent)-based model with inflow and assessed how the growth of new cases could be controlled by different interventions under different prevalence rates among visitors.

2. Methods

2.1 Individual-based model

A mass gathering event yields additional gathering occasions, called secondary gatherings, that involve local and outside populations. For an appropriate description of transmission dynamics, the import of infected people between two types of temporarily formed populations should be considered. For this reason, we employed an individual-based description of the epidemic rather than a compartmental one such as the SIR model.

Specifically, we constructed an individual-based model by extending our study of the 2009 influenza pandemic [13] to deal with main and secondary gathering events. A simulation implementing the baseline

Formatted: Highlight
Formatted: Font: Not Bold, Highlight
Formatted: Highlight
Formatted: Indent: First line: 1.72 ch
Formatted: Highlight
Field Code Changed
Formatted: Highlight
Formatted: Font: Not Bold, Highlight
Formatted: Highlight
Formatted: Font: Not Bold, Highlight
Formatted: Highlight
Commented [A2]: Expanded these sentences along with replying to Reviewer 3
Formatted: Font: Not Bold, Highlight
Formatted: Highlight
Formatted: Font: Not Bold, Highlight
Formatted: Highlight
Formatted: Font: Not Bold, Highlight
Formatted: Highlight
Formatted: Highlight
Formatted: Highlight
Formatted: Highlight
Formatted: Highlight

model describes the activity of residents in a hypothetical city consisting of five towns that are tightly connected via commuter train, each of which being a collection of households, companies, schools, and shopping centers. Temporal population in these areas are formed by role-based activity of agents, classified into students, office workers, housekeepers, and visitors from outside the city.

The state of an individual with ~~-~~respect ~~-to.~~ the disease is classified in the same manner as the SEIR model, that is, susceptible (S), infected but not yet infectious (E), infected and infectious (I), and immune (R).

~~Susceptible,~~ In each local place, susceptible agents suffer infection risk *P* during the small time period $\Delta t = 1\mathrm{min}$:

$$P = R_t \cdot \frac{\Delta t}{T_{\mathrm{inf}}} \cdot \frac{I}{N},$$

where *N* and *I* are the (temporal) numbers of total and infectious visitors, respectively, R_t is the effective reproduction number in this place, and $T_{\mathrm{inf}} = 4\mathrm{day}$ is the mean infectious period. This city is a simplified model of central Tokyo; a schematic illustration of this city is shown in Figure 1, and the numbers of entities are listed in Table 1. In total, about 1 million residents live in the city and have the chance to visit the main event, which is set to have 50,000 attendees. The timing of attendance is random.

For meanwhile, put the sentences to define the simulator at the end of the paragraph:

The main mass gathering event consists of sub-venues, each of which randomly selected residents are scheduled to attend, and attendees in different sub-events are mutually segregated.

Table 1. Populations and number of places in the simulation.

	Town 1	Town 2	Town 3	Town 4	Town 5
Population	571,641	176,866	138,684	314,861	44,680
#Shops	100	100	100	100	100
#Parks	2	2	2	2	2
#Schools	70	20	12	29	8
#Offices	100	100	100	2,000	2,000

Figure 1. Schematic illustration of the simulated city.

~~# simulated city の構成についての判断を書く~~

The simulated city is designed so that a set of places have only a threshold-level reproduction ~~number~~number, but ~~these would beare connected each other by commuter trains as well as further active an visitors~~activity

Formatted: Indent: First line: 0 ch

Formatted: Highlight (×16)

Commented [A3]: Extensively rewritten this paragraph to clarify the mechanism of our simulator

Formatted: Highlight

Formatted: Highlight

of visitors higher than residents could alter the tread in the number of new cases.

Below, we enumeratebriefly introducesummarize the assumptions on the situation in the simulated city reflected to theis design of the simulation andregarding what would happen during the event and the eir reasoning behind themits application to our simulator. Firstly, The main mass gathering event consists of sub-venues, each of which randomly selected residents are scheduled to attend, and attendees in different sub-events are mutually segregated. IiIt is unlikely that the epidemic in concern is highly reproducible not in an uptrend if when a mass gathering event is allowable. For this reason, the transmissibility parameters in populations in the simulated city have been configured so that the R_t of the entire city is nearly equal to unity and the number of daily cases is kept almost constant until the event. The game venue is also not expected to greatly modify the trend in total number of new cases.

However, a non-small number of attendees would be expected to visit some other location after the event is over in order to meet with or socialize with one another, thereby creating many secondary gathering events. Some of these gatherings cause potentially the so-called super-spreading. Game venues themselves are also not expected to directly overwrite the epidemiological trend. The remained possibility is that additional activities followed the game watching may enhance the mixing and/or cause the so-called superspreading event. Accordingly, the simulator

In simulationsTo address this possibility, game watchers in simulation are scheduled probabilistically to attend additional gathering with a variety different infection risk thereof infection to address this question..

あんまり都市内そしてゲーム会場内でトレンドを変更するほどの再生産は期待されない.

Specifically, This this calibration was carried out in a heuristic manner because unlike compartmental models, this critical condition is not described in a simple formula. In addition to a low reproducibility in general places in the citySecondly, given that Tthe prevalence among visitors is also likely to be moderately low when the event is allowed to open,

the game place itself is not expected toalso does not directly .contribute to changemodify the trend in total number of new cases.

However, Though the main game venue may be highly reproducible

Aa non-small number of attendees would be expected to visit some other location after the event is over in order to meet with or socialize with one another, thereby creating many secondary gathering events. Some of these gatherings cause potentially the so-called super-spreading.

We further assume that inbound attendees come to the main event multiple times and always join an

additional event after the main one, considering that such travelers would necessarily eat out, in contrast to local attendees. The abovementioned assumptions are reflected in the parameter setting shown in Table 2, where the infection risk is quantified in terms of the R_t. In particular, a heavy tailed distribution of Rt at secondary gathering places is introduced to describe potential superspreading events.

In households, schools, and corporations, the risk is small, unlike the 2009 swine flu pandemic. Therefore, the R_t values are set to 1. The attributed R_t value, 1.05, to places other than those for the main and sub-events was chosen heuristically to establish a sustained (non-growing) epidemic. The proportion of the main event attendees who are dropped (e.g., to meet their friends) is provided by a probabilistic distribution.

Formatted: Highlight

Formatted: Highlight

Commented [A4]: (con't)

Commented [A5R4]: Extensively rewritten this paragraph to clarify the mechanism of our simulator

Formatted: Highlight

(removed sentences)

Table 2. Parameters defining the activity of people in the simulation.

Reproduction number ($\mathbf{R}_t$)			
	Main events	25 (baseline) or 1 (controlled)	
	Secondary events (uncontrolled)*	17-24 (90%), 240 (10%)	
	Secondary events (controlled)*		
	Other sites	1	
		Domestic	Inbound
Prevalence		0.01%	≦1% (secenario-dependent)
Per capita attendance times		1	1-3
Probability of a seconcary event*		0-0.1 (50%), 0.1-0.9 (40%), 0.9-1 (10%)	1

2.2 Scenarios

To examine the possibility of suppressing the additional impetus to the epidemic brought by visitors, we carried out simulations with the following three scenarios:

1. **StadiumOnly:** The control is administered only in the main event venue. In the simulation, the control is implemented by a reduction in the R_t from 25 to 1 (see Table 2).
2. **Extra:** In addition to StadiumOnly, secondary events are assumed to be controlled. This is implemented by reducing the proportion of highly risky places (R_t = 240) for secondary events from 10% to 1%.
3. **WithoutDrop:** In addition to StadiumOnly, most of the attendees (specifically 90% in the simulation) are assumed to go directly home in accordance with a corresponding governmental campaign. The risk distribution in secondary events is the same as that in the StadiumOnly scenario.

The abovementioned values are arbitrary, but reflect the following assessment. First, in the simulations, we set the latent period to 3.5 days and the infectious period to 3 days, as average. We here assign rather shorter values, considering these periods are exponentially distributed in our model while the reality is close to a gamma or Weibull distribution [14]. Second, Murakami [15] assessed the infection risk in the Opening Ceremonies of the Tokyo 2020 Olympic Games as follows. An infectious attendee would yield about 1.5 or 0.01 new cases in uncontrolled and controlled settings, respectively, during a 5-hour-long ceremony, which is translated into the $\mathbf{R}_t$ by:

$$\mathbf{R}_t = (3 \text{ days})/(5 \,/\, 24 \text{ days}) \times (1.5 \text{ or } 0.01) \text{ per capita cases} \approx 22 \text{ or } 0.1.$$

As for the controlled case, we used the threshold value, R_t = 1, instead for this rather small risk. A similar

Formatted: Highlight

Formatted: Highlight

Formatted: Highlight

Formatted: Highlight

assessment is available for another event, a 4-hour long soccer game: 0.74 or 0.022 cases are reproduced by one infectious case, respectively. A higher risk at some of the secondary gatherings is based on a survey targeting a large indoor convention [16]. That study showed that respondents testing positive were more likely to have attended pubs, karaoke bars, or nightclubs. Using their reported values, attendees to these additional activities are at roughly three times higher risk of infection. Further assuming that this tripled risk is experienced within 1.5 hours, we take $\mathrm{R}_t = 24 \times 5/1.5 \times 3 = 240$ to be the worst case scenario at additional meetings.

Formatted: Highlight

3. Results

Whether main mass gathering events accelerate epidemics depends on the baseline setting, the prevalence rate among visitors, and possible counter-measures to help prevent close contact. Figure 2 summarizes the outcome under each setting as an epidemic curve. As a baseline, the transmissibility parameters in respective small populations in the simulated city were configured so that the R_t of the entire city remained nearly equal to unity until the event opened. As was configured, the number of new cases remained almost constant until the main event opened on day 14, and then tended to increase with a different slope until the event closed on day 24.

With the measure against only the event venue (scenario **StadiumOnly**) and zero infectious visitors, the new cases increased with a slope of R_t = 1.08, as shown at the top left of Figure 2. This increasing trend was sustained when the prevalence of infection among the visitors was mildly high, $p_{\mathrm{inf,vis}} = 0.1\%$, but rapidly increased to R_t = 1.33 when the prevalence of infection was extremely high, at $p\_(\mathrm{inf,vis}) = 1\%$. These results suggest that cases imported from the outside would have only a minor impact if $p_{\mathrm{inf,vis}} < 0.1\%$.

Regarding secondary events under scenario **Extra**, it may be possible to erase the uptrend attributable to the main event when the visitors are fully noninfectious. Infectious visitors may reinstate an uptrend, but our findings suggest that it would be rather weak as long as the prevalence was mild: R_t = 1.04 for $p\_(\mathrm{inf,vis}) = 0.1\%$. A recommendation to visitors to return directly home (scenario **WithoutDrop**) would have a similar effect. A constant trend would be sustained after the start of the event start if no infectious visitors came in, or a shift to a weak uptrend if their prevalence rate was <0.1%.

So far, we have interpreted the impacts of infectious visitors and controls, viewing only the mean epidemic curve trajectories. However, these curves are highly stochastic, as shown in the gray shading in each panel of Figure 2, and the slope of the trend involves a greater uncertainty, as shown in Figure 3. Figure 4 shows the R_t with an uncertainty for all scenarios as a function of $p_{\mathrm{inf,vis}}$. The strongest measure of **WithoutDrop** suppresses the increase in the R_t based on the prevalence rate of visitors. On average, $p\_(\mathrm{inf,vis}) \leq 0.1\%$ keeps the R_t almost at unity under this measure.

Figure 2. Simulated epidemic curves for different assumptions on the prevalence rate among visitors and possible measures. Thick lines indicate the mean (blue) and median (red) trajectories, while thin lines correspond to respective quantiles (25th, 75th, 80th, 85th, 90th, and 95th percentiles). The main mass gathering event runs from day 14 through 23 and is bounded by vertical lines.

Figure 3. The effective reproduction number (R_t) in the entire city during the main event in each scenario. The uncertainty in each setting is evaluated from 1,000 runs with a different random number sequence.

Figure 4. Distributions of cumulative cases during the event corresponding to Figure 2. The uncertainty is based on 1,000 simulation runs.

4. Discussion and Conclusion

In this study, we demonstrated how the potential growth of an epidemic can be successfully or unsuccessfully suppressed in a mass gathering event involving a comparable number of local and outside visitors depending on the strength of the intervention and the prevalence rate among visitors. As a general remark, the growth rate potentially enhanced by hosting a mass gathering can be suppressed to a level identical or slightly higher than that of the baseline if the places for gathering after the main event are well controlled and the prevalence rate among visitors is mild relative to that among local residents.

~~{~~

The controls discussed in this work are voluntary-basis and hence ~~their effectiveness is limited by depends on how~~the degree people~~the participants~~ are willing to cooperate limits the effectiveness. A massive superspreading along with Kumpha Mela [11] religious could be numbered as a case that such a cooperation is poorly established, and many attendees reportedly did not follow to the requests of mask wearing and social distancing. Though we are not sure how to measure directly such a willingness via investigation, we can refer some articles which help future discussion. People yielding higher score in willingness to cooperate may not show a greater acceptance of behavioral regulations [17~~article 1~~]. The cultural difference affects on which of individual-basis or law-enforced measures are preferable. A comparative study of three countries [18~~article 2~~] shows Chinese participants are more acceptable to enforced measures than Japan and US, while US and Chinese more accept individual-level measures than does Japanese. Another investigation [19~~article 3~~] supports that Japanese people are reluctant to enforced restrictions in a greater degree than the other participating countries. As for mask wearing, one of self-motivated measures, there are studies over 50 US states and over the world that support people in collectivistic communities more frequently wear masks than in individual ones [20~~article 5~~]. Also, it is possible to build a cooperative relationship among supports fan, academic experts, and event organizers: as high as about 98% of chant cheering participants wore masks in games held by Japan Football leagues [21~~article 4~~].

~~}~~

Formatted: Highlight
Formatted: Indent: First line: 1.35 ch
Formatted: Indent: First line: 1.35 ch
Formatted: Highlight
Formatted: Highlight
Formatted: Font: Not Bold, Highlight
Formatted: Font: Not Bold, Highlight
Formatted: Font: Not Bold, Highlight
Formatted: Highlight
Formatted: Font: Not Bold, Highlight
Formatted: Font: Not Bold, Highlight
Formatted: Font: Not Bold, Highlight
Formatted: Highlight
Formatted: Font: Not Bold, Highlight
Formatted: Font: Not Bold, Highlight
Formatted: Font: Not Bold, Highlight
Formatted: Highlight
Formatted: Font: Not Bold, Highlight
Formatted: Font: Not Bold, Highlight
Formatted: Font: Not Bold, Highlight
Formatted: Highlight
Formatted: Font: Not Bold, Highlight
Formatted: Font: Not Bold, Highlight
Formatted: Font: Not Bold, Highlight
Formatted: Highlight

Commented [A6]: Revised to reply Reviewer 3's comments for the limitation of proposed method

Reflecting the lack of qualitatively interpretable data for shops, restaurants, and other kinds of possible meeting places, we carried out simulations with scenario-based settings to assess risk in these places. Below, we discuss the availability of data from the parameters of our simulation.

The Japanese government enacted a strict quarantine at airports during the pandemic. The data collected from the quarantine [22,23] could be expected to aid the estimation of prevalence rates among visitors, thereby making quarantine more effective in combination with testing before boarding and after arrival [24].

In addition to the prevalence obtained along with the quarantine, the prevalence rate among the participants of the Tokyo 2020 Olympic Games is available [25]. Maintaining a sufficient level of control at secondary gathering places is more difficult than the main event venue. The Tokyo Metropolitan Government issued an "Infection Prevention Thorough Declaration Sticker" to shops, restaurants, and other meeting places to establish a certain level of risk reduction and provide information on well-sanitized places to local residents [26]. Hence, the ratio of safe places to 'risky places' (10% or 1% being assumed in the present study) may be determined via data. However, the requirements for this are mainly qualitative, and it is nontrivial to translate these data into the risk of infection computationally interpretable.

Observational facts that contribute to connecting the emission of droplets to the risk of infection have accumulated since the beginning of the COVID-19 pandemic and the abovementioned difficulties may be overcome by plugging these data into simulations. While some of this knowledge has yet to be fully authorized, a number of studies have already been published. Ando et al. [27] assessed infection risk at a restaurant using a dedicated hydrodynamical model on the Fugaku supercomputer. The effects of air ventilation in the venue have been monitored based on CO_2 concentrations during a football game [28], and droplet emissions have been measured during normal speech [29], coughing [30], and singing [31]. In addition, a COVID-19 outbreak event suspected to be induced by air conditioning in a restaurant has been reported [32]. We expect that a reliable set of infection risk parameters will become available based on the outcomes of empirical studies interpreted inside hydrodynamical droplet simulations, and that individual-based models will contribute to improved risk assessments against new variants of severe acute respiratory syndrome coronavirus 2 and future pandemics caused by other infectious diseases.

Formatted: Highlight

Formatted: Highlight

Formatted: Highlight

Formatted: Highlight

Formatted: Highlight

Formatted: Highlight

Formatted: Highlight

~~Acknowledgements~~

~~This work was supported by JSPS KAKENHI Grant Numbers JP18K11541.~~

References

1. Jones RM. Relative contributions of transmission routes for COVID-19 among healthcare personnel providing patient care. J. Occup. Environ. Hyg. 2020;17: 408–415.
2. Nicas M, Sun G, 2006. An integrated model of infection risk in a health-care environment. Risk Anal 2006; 26: 1085–1096.
3. Riley S. Large-Scale Spatial-Transmission Models of Infectious Disease. Science 2007;316: 1298-1301. doi: 10.1126/science.1134695.
4. Ferguson NM et al. Strategies for containing an emerging influenza pandemic in Southeast Asia. Nature

2005;437: 209-14. doi: 10.1038/nature04795.

5. Longini IM, Jr. et al. Containing Pandemic Influenza at the Source. Science 2005; 309: 1083-87.

6. Aleta A, Martin-Corral D, Pastore y Piontti A, Ajelli M, Litvinova M, Chinazzi M, Dean NE, Halloran ME, Longini IM Jr, Merler S, Pentland A, Vespignani A, Moro E, Moreno Y. Modelling the impact of testing, contact tracing and household quarantine on second waves of COVID-19. Nature Human Behaviour 2020; 4: 964-971.

~~[cowling2015]~~ Cowling BJ, Park M, Fang VJ, Wu P, Leung GM, Wu JT. Preliminary epidemiological assessment of MERS-CoV outbreak in South Korea, May to June 2015. Euro Surveill. 2015;20(25):pii=21163.
Available online: http://www.eurosurveillance.org/ViewArticle.aspx?ArticleId=21163

7. ~~[nishiura2015]~~ Nishiura H, Endo A, Saito MM, Kinoshita R, Ueno R, Nakaoka S, Miyamatsu Y et al. Identifying determinants of heterogeneous transmission dynamics of the Middle East respiratory syndrome (MERS) outbreak in the Republic of Korea, 2015: a retrospective epidemiological analysis. BMJ Open. 2016;6:e009936. doi: 10.1136/bmjopen-2015-009936

8. ~~[reviewer 3-1]~~ Mai NL, Alhady STM, Nguyen TMD, Le VT, Qarawi A, Venkatesh U, et al. Evaluation of risk factors associated with SARS-CoV-2 transmission, Current Medical Research and Opinion. Current Medical Research and Opinion. 2022; 38(12), 2021-2028. doi: 10.1080/03007995.2022.2125258

9. Son WS, RISEWIDs Team. Individual-based simulation model for COVID-19 transmission in Daegu, Korea. Epidemiology and Health 2020; 42: e2929942.

10. ~~[reviewer 3-2]~~ Rocha ICN, Pelayo MGA, Rackimuthu S. Kumbh Mela Religious Gathering as a Massive Superspreading Event: Potential Culprit for the Exponential Surge of COVID-19 Cases in India. Am. J. Trop. Med. Hyg. 2021;105(4), 868-871. doi: 10.4269/ajtmh.21-0601

11. ~~[Reviewer 3-3]~~ Rocha ICN, Pelayo MGA, Sammatid C. Traveling and Celebrating During Songkran as Super Spreading Events: A Potential Triggering Factor of the Surge of COVID-19 Cases in Thailand. Int J Travel Med Glob Health. 2021;9(4):196-197. doi 10.34172/ijtmgh.2021.33

~~7.~~12.

~~8.~~13. Saito MM et al. Enhancement of collective immunity in Tokyo Metropolitan area by selective vaccination against an emerging influenza pandemic. PLOS ONE 2013; 8: e72866. doi: 10.1371/journal.pone.0072866.

~~9.~~14. Ferguson N et al. Report 9: Impact of non-pharmaceutical interventions (NPIs) to reduce COVID19

Formatted: Highlight

Formatted: List Paragraph, Numbered + Level: 1 + Numbering Style: 1, 2, 3, … + Start at: 1 + Alignment: Left + Aligned at: 0" + Indent at: 0.29"

Formatted: Font: Not Bold, Highlight

Formatted: Highlight

Formatted: Font color: Text 1, Highlight

Formatted: Highlight

Formatted: Font color: Text 1, Highlight

Formatted: List Paragraph, Numbered + Level: 1 + Numbering Style: 1, 2, 3, … + Start at: 1 + Alignment: Left + Aligned at: 0" + Indent at: 0.29"

Formatted: Highlight

Formatted: List Paragraph, Numbered + Level: 1 + Numbering Style: 1, 2, 3, … + Start at: 1 + Alignment: Left + Aligned at: 0" + Indent at: 0.29"

Commented [A7]: Newly referred articles

Formatted: Highlight

Formatted: Font color: Text 1, Highlight

mortality and healthcare demand. 2020. doi: 10.25561/77482.

10.15. Murakami et al. COVID-19 risk assessment at the opening ceremony of the Tokyo 2020 Olympic Games. Microbial Risk Analysis 2020; 19: 100162. doi: 10.1016/j.mran.2021.100162.

16. Sami S et al. 2022. Investigation of SARS-CoV-2 Transmission associated with a large indoor convention — New York City, November–December 2021. Morbidity and Mortality Weekly Report 2022; 71(7): 243-248. doi: http://dx.doi.org/10.15585/mmwr.mm7107a4

17. [artcile1] Zhu N, Smetana JG, Chang L. Acceptance of Society-Level and Individual-Level Preventive Measures during the COVID-19 Pandemic among College Students in Three Societies. Journal of Cross-Cultural Psychology 2021; 52(7): 606–621. https://journals.sagepub.com/doi/10.1177/00220221211995971?icid=int.sj-full-text.similar-articles.5

18. [article 2] Romano A, Spadaro G, Balliet D, Joireman J, van Lissa C, Jin S, Agostini M, Bélanger JJ, Gützkow B, Kreienkamp J, Leander NP. Cooperation and Trust Across Societies During the COVID-19 Pandemic. Journal of Cross-Cultural Psychology 2021; 52(7):622–642. https://journals.sagepub.com/doi/full/10.1177/0022022120988913

19. [article 3] Murakami M, Hiraishi K, Yamagata M, Nakanishi D, Ortolani A, Mifune N, Li Y, Miura A. 2023. Differences in and associations between belief in just deserts and human rights restrictions over a 3-year period in five countries during the COVID-19 pandemic. PeerJ 2023; 11:e16147 http://doi.org/10.7717/peerj.16147

20. [article 5] Lu JG, Jin P, English AS. Collectivism predicts mask use during COVID-19. PNAS 2021;118(23). https://doi.org/10.1073/pnas.2021793118

11.21. [article 4] Yasutaka T, Onishi M, Naito W, Bando Y, Okuda T. Maximum proportion of masks worn: Collaborative efforts in Japanese professional football. Journal of Infection and Public Health 2023. https://doi.org/10.1016/j.jiph.2023.03.009

12.22. Ministry of Health, Labour and Welfare (MHLW). Press release of COVID (domestic new cases and quarantine at air- and seaports; in Japanese). 2021. Available from: https://www.mhlw.go.jp/stf/seisakunitsuite/bunya/0000121431_00254.html

13.23. Tokyo Metropolitan Government. Tokyo Metropolitan Government COVID-19 monitoring meeting minutes (in Japanese). 2020. Available from: URL: https://www.bousai.metro.tokyo.lg.jp/taisaku/saigai/1009676/index.html

14.24. Kamo M, Murakami M, Imoto S. Effects of test timing and isolation length to reduce the risk of COVID-19 infection associated with airplane travel, as determined by infectious disease dynamics modeling. Microbial Risk Analysis 2022;20: 1-8. doi: 10.1016/j.mran.2021.100199.

15.25. Tokyo Metropolitan Government. The values Tokyo metropolitan brought to us (in Japanese). 2021. Available from: https://www.2020games.metro.tokyo.lg.jp/special/1b340dde24650e2db8121738cef945cf.pdf

16.26. Tokyo Metropolitan Government. Infection Prevention Thorough Declaration Sticker (in Japanese). 2021.

Formatted: Highlight

Commented [A8]: Newly referred articles

Formatted: Highlight

Formatted: No underline, Font color: Auto, Highlight

Formatted: Highlight

Formatted: No underline, Font color: Auto, Highlight

Formatted: Highlight

Available from: https://web.archive.org/web/20230404213919/https://www.bousai.metro.tokyo.lg.jp/taisaku/torikumi/1008262/1008420/index.html~~https://www.bousai.metro.tokyo.lg.jp/taisaku/torikumi/1008262/1008420/index.html~~

~~17.~~27. Ando K, Bale R, Li CG, Matsuoka S, Onishi K, Tsubokura M. Digital transformation of droplet/aerosol infection risk assessment realized on "Fugaku" for the fight against COVID-19. to appear on International Journal of High-Performance Computing Application, to appear (preprint is available from arXiv:2110.09769v3).

~~18.~~28. Murakami M, Yasutaka T, Onishi M, Naito W, Shinohara N, Okuda T, Fujii K, Katayama K, Imoto, S. Living with COVID-19: mass gatherings and minimizing risk. QJM: An International Journal of Medicine 2021; 114 (7): 437–439. doi:10.1093/qjmed/hcab163.

~~19.~~29. Asadi S, Wexler AS, Cappa CD, Barreda S, Bouvier NM, Ristenpart WD. Aerosol emission and superemission during human speech increase with voice loudness. Scientific Reports 2019; 9:2348. doi:10.1038/s41598-019-38808-z

~~20.~~30. Milton DK, Fabian MP, Cowling BJ, Grantham ML, McDevitt JJ. Influenza Virus Aerosols in Human Exhaled Breath: Particle Size, Culturability, and Effect of Surgical Masks. PLoS Pathog 2013;9(3): e1003205. doi:10.1371/journal.ppat.

~~21.~~31. Alsveda M, Matamisb A, Bohlinc R, Richterb M, Bengtssonb PE, Fraenkeld CJ, Medstrande P, Londahl J. Exhaled respiratory particles during singing and talking. Aerosol Scicence and Technology 2020;54(11): 1245–1248. doi: 10.1080/02786826.2020.1812502.

~~22.~~32. Lu J, Gu J, Li K, Xu C, Su W, Lai Z, Zhou D, Yu C, Xu B, Yang Z. COVID-19 Outbreak Associated with Air Conditioning in Restaurant, Guangzhou, China, 2020. Emerging Infectious Diseases 2020; 26(7): 1628-31.

Formatted: Highlight

Commented [A9]: Obsoleted url is updated

Formatted: Highlight

Response to Reviewers

Reply to Reviewer 1

Could you please provide further detail on **how the individual based simulator was built** and the specific assumptions used in the model?
Can you elaborate on the **reasons behind the choice of the hypothetical city** used in the simulation?

[answer] Our main interest is to assess whether additional gathering accompanying the main event enhance the reproduction. Emphasizing this point, we have rephrased the paragraphs in section 2.1 Individual-based model.

Were any limitation or assumptions made regarding the potential spread of the infectious disease beyond the event and the city?

[answer] In this study, we haven't discussed in detail how degree people are willing to corporate the recommendation. Along with your next remark, we have add a discussion section for this topic.

While the results of your study suggest that the increase in the number of COVID-19 patients can be suppressed to a level equivalent to that if the event were not being held, can you discuss any potential challenges or limitations to implementing the suggested controls and restrictions on visitors?

[answer] The controls discussed in this work are voluntary-basis and their effectiveness depends on how degree the participants are willing to cooperate. We refer several articles which do not directly evaluate this but would help us to discuss this problem and add the sentences below:

- The controls discussed in this work are voluntary-basis and hence the degree people are willing to cooperate limits the effectiveness. People yielding higher score in willingness to cooperate may not show a greater acceptance of behavioral regulations [17].
- The cultural difference affects on which of individual-basis or law-enforced measures are preferable. A comparative study of three countries [18] shows Chinese participants are more acceptable to enforced measures than Japan and US, while US and Chinese more accept individual-level measures than does Japanese.

Another investigation [19] supports that Japanese people are reluctant to enforced restrictions in a greater degree than the other participating countries.

- As for mask wearing, one of self-motivated measures, there are studies over 50 US states and over the world that support people in collectivistic communities more frequently wear masks than in individual ones [20].
- Also, it is possible to build a cooperative relationship among supports fan, academic experts, and event organizers: as high as about 98% of chant cheering participants wore masks in games held by Japan Football leagues [21].

Replay to Reviewer 3

---

2) A mass gathering event can be superspreading events (SSE) and it has a fairly formal definition in infectious disease epidemiology literature, first requiring an estimate of R, and then identifying events where the number of secondary infections from a single case is substantially above the average (i.e. substantially above R), a minimum threshold might be at least 2x R or 3x R. In this regard, I would like you to read on this paper and cite to further improve the introduction and discussion sections,
https://doi.org/10.1080/03007995.2022.2125258

3) Additionally, the authors may also use these papers in describing and citing SSEs from other countries, such as what happened in India (the Kumbh Mela massive SSE which infected millions of people) https://doi.org/10.4269/ajtmh.21-0601 and in Thailand (the scattered SSEs during holidays) https://doi.org/10.34172/ijtmgh.2021.33

[answer] Thank you for giving a remark on the importance of superspreading and related references. To address your suggestion, we have added a topic addressing your suggestion on superspreading in Introduction.

---

4) It is also nice to include some recommendations and to further improve the conclusion.

[answer] I have added a paragraph in Conclusion section to discuss how degree voluntary based measures are accepted. The Kumbh Mela massive SSE case is again referred here as a case study where a cooperation among participants is not well established.

Changes in reference list

I below show the changes in the reference list. The number to each reference is of the present version (i.e. renumbered from the original submission).

- Added articles in this revision: 7, 8, 9, 10, 11, 12, 17, 18, 19, 20, and 21.
- Removed articles in this revision: none.
- Articles whose reference is modified: 26
  - Reason: The original URL in a governmental web site is no longer available and replaced to wayback machine's URL.
