# Supplementary material for: Effects of inbound attendees of a mass gathering event on the COVID-19 epidemic using individual-based simulations
Source: PLoS One. 2025 Apr 23;20(4):e0321288. doi: 10.1371/journal.pone.0321288 (PMC12017503; doi:10.1371/journal.pone.0321288)
Supplement: Attachment — Submitted filename: 505-08-30_reply_to_comment.docx [file pone.0321288.s001.docx]

**Response to Reviewers**

**Reply to Reviewer 1**

Could you please provide further detail on **how the individual based simulator was built** and the specific assumptions used in the model?

Can you elaborate on the **reasons behind the choice of the hypothetical city** used in the simulation?

**[answer]** Our main interest is to assess whether additional gathering accompanying the main event enhance the reproduction. Emphasizing this point, we have rephrased the paragraphs in section 2.1 Individual-based model.

Were any limitation or assumptions made regarding the potential spread of the infectious disease beyond the event and the city?

**[answer]** In this study, we haven’t discussed in detail how degree people are willing to corporate the recommendation. Along with your next remark, we have add a discussion section for this topic.

While the results of your study suggest that the increase in the number of COVID-19 patients can be suppressed to a level equivalent to that if the event were not being held, can you discuss any potential challenges or limitations to implementing the suggested controls and restrictions on visitors?

**[answer]** The controls discussed in this work are voluntary-basis and their effectiveness depends on how degree the participants are willing to cooperate. We refer several articles which do not directly evaluate this but would help us to discuss this problem and add the sentences below:

- The controls discussed in this work are voluntary-basis and hence the degree people are willing to cooperate limits the effectiveness. People yielding higher score in willingness to cooperate may not show a greater acceptance of behavioral regulations **[17]**.
- The cultural difference affects on which of individual-basis or law-enforced measures are preferable. A comparative study of three countries **[18]** shows Chinese participants are more acceptable to enforced measures than Japan and US, while US and Chinese more accept individual-level measures than does Japanese. Another investigation **[19]** supports that Japanese people are reluctant to enforced restrictions in a greater degree than the other participating countries.
- As for mask wearing, one of self-motivated measures, there are studies over 50 US states and over the world that support people in collectivistic communities more frequently wear masks than in individual ones **[20]**.
- Also, it is possible to build a cooperative relationship among supports fan, academic experts, and event organizers: as high as about 98% of chant cheering participants wore masks in games held by Japan Football leagues **[21]**.

**Replay to Reviewer 3**

2) A mass gathering event can be superspreading events (SSE) and it has a fairly formal definition in infectious disease epidemiology literature, first requiring an estimate of R, and then identifying events where the number of secondary infections from a single case is substantially above the average (i.e. substantially above R), a minimum threshold might be at least 2x R or 3x R. In this regard, I would like you to read on this paper and cite to further improve the introduction and discussion sections,
<https://doi.org/10.1080/03007995.2022.2125258>

3) Additionally, the authors may also use these papers in describing and citing SSEs from other countries, such as what happened in India (the Kumbh Mela massive SSE which infected millions of people) <https://doi.org/10.4269/ajtmh.21-0601> and in Thailand (the scattered SSEs during holidays) <https://doi.org/10.34172/ijtmgh.2021.33>

**[answer]** Thank you for giving a remark on the importance of superspreading and related references. To address your suggestion, we have added a topic addressing your suggestion on superspreading in Introduction.

4) It is also nice to include some recommendations and to further improve the conclusion.

**[answer]** I have added a paragraph in Conclusion section to discuss how degree voluntary based measures are accepted. The Kumbh Mela massive SSE case is again referred here as a case study where a cooperation among participants is not well established.

**Changes in reference list**

I below show the changes in the reference list. The number to each reference is of the present version (i.e. renumbered from the original submission).

- Added articles in this revision: 7, 8, 9, 10, 11, 12, 17, 18, 19, 20, and 21.
- Removed articles in this revision: none.
- Articles whose reference is modified: 26
  - Reason: The original URL in a governmental web site is no longer available and replaced to wayback machine’s URL.
